# *Agrobacterium*-mediated vacuum infiltration and floral dip transformation of rapid-cycling *Brassica rapa*

**DOI:** 10.1186/s12870-019-1843-6

**Published:** 2019-06-10

**Authors:** Die Hu, Andrew F. Bent, Xilin Hou, Ying Li

**Affiliations:** 1 National Key Laboratory of Crop Genetics and Germplasm Enhancement, College of Horticulture, Nanjing Agricultural University, Nanjing, 210095 Jiangsu Province China; 2 Department of Plant Pathology, University of Wisconsin–Madison, Madison, WI 53706 USA

**Keywords:** Rapid-cycling *Brassica rapa*, Wisconsin fast plants, *Agrobacterium*-mediated transformation, Vacuum infiltration, Floral dip

## Abstract

**Background:**

Rapid-cycling *Brassica rapa* (RCBr), also known as Wisconsin Fast Plants, are small robust plants with a short lifecycle that are widely used in biology teaching. RCBr have been used for decades but there are no published reports of RCBr genetic transformation. *Agrobacterium*-mediated vacuum infiltration has been used to transform pakchoi (*Brassica rapa* ssp. *chinensis*) and may be suitable for RCBr transformation. The floral dip transformation method, an improved version of vacuum infiltration, could make the procedure easier.

**Results:**

Based on previous findings from *Arabidopsis* and pakchoi, plants of three different ages were inoculated with *Agrobacterium*. Kanamycin selection was suboptimal with RCBr; a GFP screen was used to identify candidate transformants. RCBr floral bud dissection showed that only buds with a diameter less than 1 mm carried unsealed carpels, a key point of successful floral dip transformation. Plants across a wide range of inflorescence maturities but containing these immature buds were successfully transformed, at an overall rate of 0.1% (one per 1000 T_1_ seeds). Transformation was successful using either vacuum infiltration or the floral dip method, as confirmed by PCR and Southern blot.

**Conclusion:**

A genetic transformation system for RCBr was established in this study. This will promote development of new biology teaching tools as well as basic biology research on *Brassica rapa*.

**Electronic supplementary material:**

The online version of this article (10.1186/s12870-019-1843-6) contains supplementary material, which is available to authorized users.

## Background

Rapid-cycling *Brassica rapa* (RCBr), also known as Wisconsin Fast Plants, were derived from genetic crossing among multiple faster flowering *Brassica rapa* [1]. Because of this origin, RCBr inherited diverse traits. Equally important, the plants are petite and have a very short lifecycle of about 40 days if grown under continuous light. Due to these desirable characteristics, and the development of clever and inexpensive teaching modules by the Wisconsin Fast Plants program, RCBr have been used in thousands of classrooms to educate students about biology [2]. Scientists have also utilized RCBr for diverse studies [3–7], and RCBr is a useful model for further study of *Brassica rapa* or of plants in general.

Genetic transformation is an important tool in molecular biology research and agricultural biotechnology application. For plants, *Agrobacterium tumefaciens* is widely used for gene delivery and tissue culture is commonly utilized to generate transformants. *Brassica rapa* is thought to be recalcitrant to in vitro shoot regeneration [8] and genetic transformation of *Brassica rapa* is challenging, requiring specialist approaches and yielding success at very low frequencies [9]. Genetic background differences among cultivars play an important role in *Brassica rapa* transformation efficiency [9]. Regeneration protocols for RCBr are available [10–12]. However, genetic transformation of RCBr has not been reported. Development of a transformation system would promote both basic research on *Brassica rapa* and enhanced use of RCBr as a teaching tool.

*In planta* plant transformation methods, such as vacuum infiltration of *Agrobacterium*, are an alternative approach to plant transformation [13, 14]. Compared to tissue culture, such methods are typically less time-consuming and labor-intensive [15]. Confirmed successes with *in planta* transformation had previously been limited to plants in the Brassicaceae, but successes have now been reported for *Eustoma grandiflorum*, *Setaria viridis* and *Solanum lycopersicum* [16–18]. Pakchoi (*Brassica rapa* ssp. *chinensis*, also called bok choy or non-heading Chinese cabbage) has been transformed via *Agrobacterium*-mediated vacuum infiltration method [15, 19], suggesting the feasibility of adopting the method for RCBr transformation. The floral dip method, an improved version of vacuum infiltration [20], has been widely adopted for transformation of *Arabidopsis thaliana*, offering a simple and inexpensive approach that has a high success rate. Because the vacuum infiltration process is unnecessary to transform *Arabidopsis* [14, 20], the viability of floral dip for transformation for the taxonomically related RCBr also merits investigation.

In this study, we successfully transformed RCBr using *Agrobacterium* vacuum infiltration and floral dip methods. We found that RCBr floral buds with a diameter less than 1 mm offer the most likely target for inoculation with *Agrobacterium*. A GFP marker was used in preference to kanamycin selection to identify candidate transformants. GFP-positive progenies were obtained from plants of three different ages via these two *in planta* methods. Transformation efficiencies were not significantly different among the treatment groups. PCR and Southern blot assays verified transformation, the availability of which opens numerous opportunities for expanded use of RCBr.

## Results

### Selectable marker

**Fig. 1:**
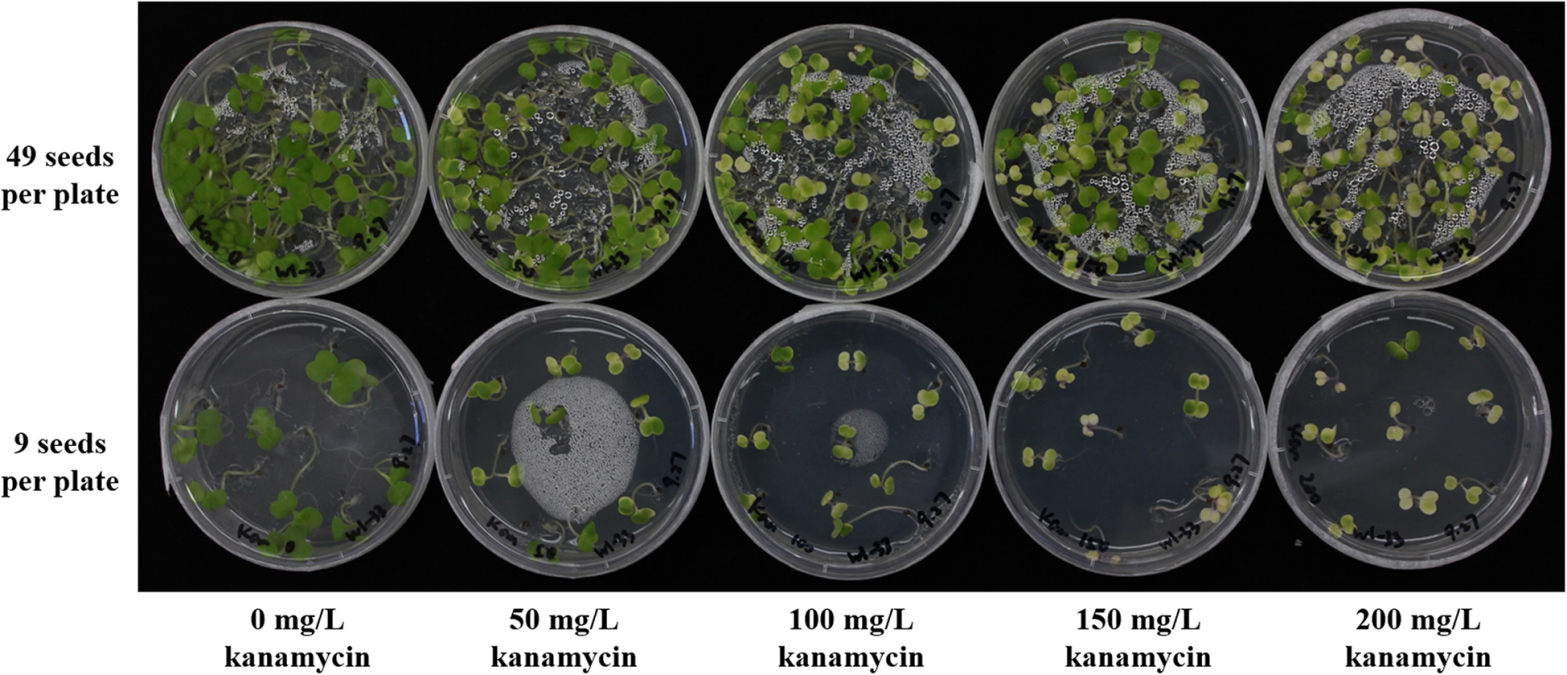
Kanamycin selection on wild-type rapid-cycling *Brassica rapa*. The diameters of presented plates were 90 mm. The image was taken 12 days after plating

An appropriate selectable or screenable marker is a crucial component of genetic transformation protocols. In most plant transformation systems, an antibiotic or herbicide resistance gene or fluorescent protein gene is used. To identify a useful method for a rapid-cycling *Brassica rapa* (RCBr) transformation system, kanamycin resistance and green fluorescent protein (GFP) expression were tested on RcBC 1-33, the standard Wisconsin Fast Plants line of RCBr. Seeds were surface-sterilized and plated onto half strength MS media containing 4 different concentrations of kanamycin. After 12 days, green seedlings were still found in plates with 200 mg/L kanamycin, even when seeds were plated at a low density (Fig. 1). For GFP expression testing, sterilized wild-type seeds were plated onto half strength MS media. About 4 days later, over 100 seedlings were screened under a fluorescence stereomicroscope and none of them contained GFP signal (see Additional file 2). These results suggested that RCBr is relatively kanamycin-insensitive and that GFP would be a better selection marker than kanamycin resistance in a RCBr transformation system.

### Bud dissection to determine inoculation stage

**Fig. 2:**
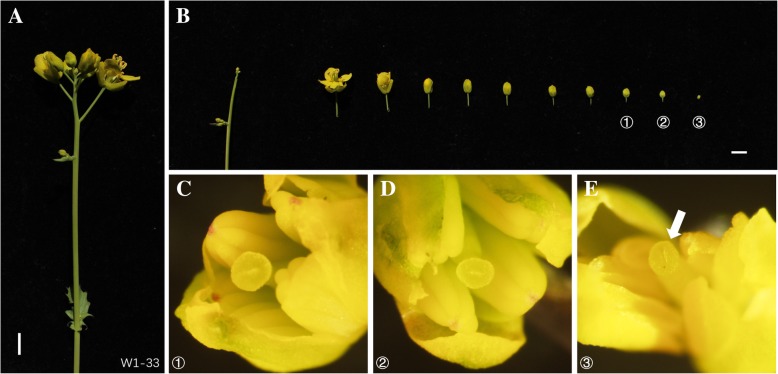
Bud dissection of rapid-cycling *Brassica rapa*. **a** Intact inflorescence of RcBC 1-33, the standard line of RCBr, prior to dissection. **b** Floral buds from one inflorescence placed in order of occurrence on the stem. Scale bars 5 mm. **c**, **d** and **e**, Dissection of indicated floral buds from B showing: ① and ②, sealed carpels, diameters of buds were around 2 mm. ③, unsealed carpel (white arrow), the diameter of bud was less than 1 mm

Because ovules are the target of *Agrobacterium*-mediated vacuum infiltration or floral dip transformation of *Arabidopsis* [21–23] and only young flowers with unclosed locules are transformable by these methods [23], flower bud dissection was performed to assess the RCBr bud growth stages most likely to yield successful transformants. Inflorescences with one or two open flowers were chosen and multiple floral buds were then dissected. Only buds with a diameter less than 1 mm had an unsealed carpel due to an incompletely formed stigma, while locules of buds with diameters around 2 mm appeared to be closed (Fig. 2). Unlike *Arabidopsis*, only a limited subset of RCBr flowers form seed pods and application of *Agrobacterium* to flower buds with closed locules is likely to be unproductive [23]. As an added consideration, removing flowers or older buds requires additional labor and can destroy the whole inflorescence. The above findings suggested that application of *Agrobacterium* to RCBr might best be done at a relatively early stage of inflorescence development, for example when the oldest flower buds still have their petals mostly enclosed by sepals.

### Transformation by vacuum infiltration or floral dip

**Fig. 3:**
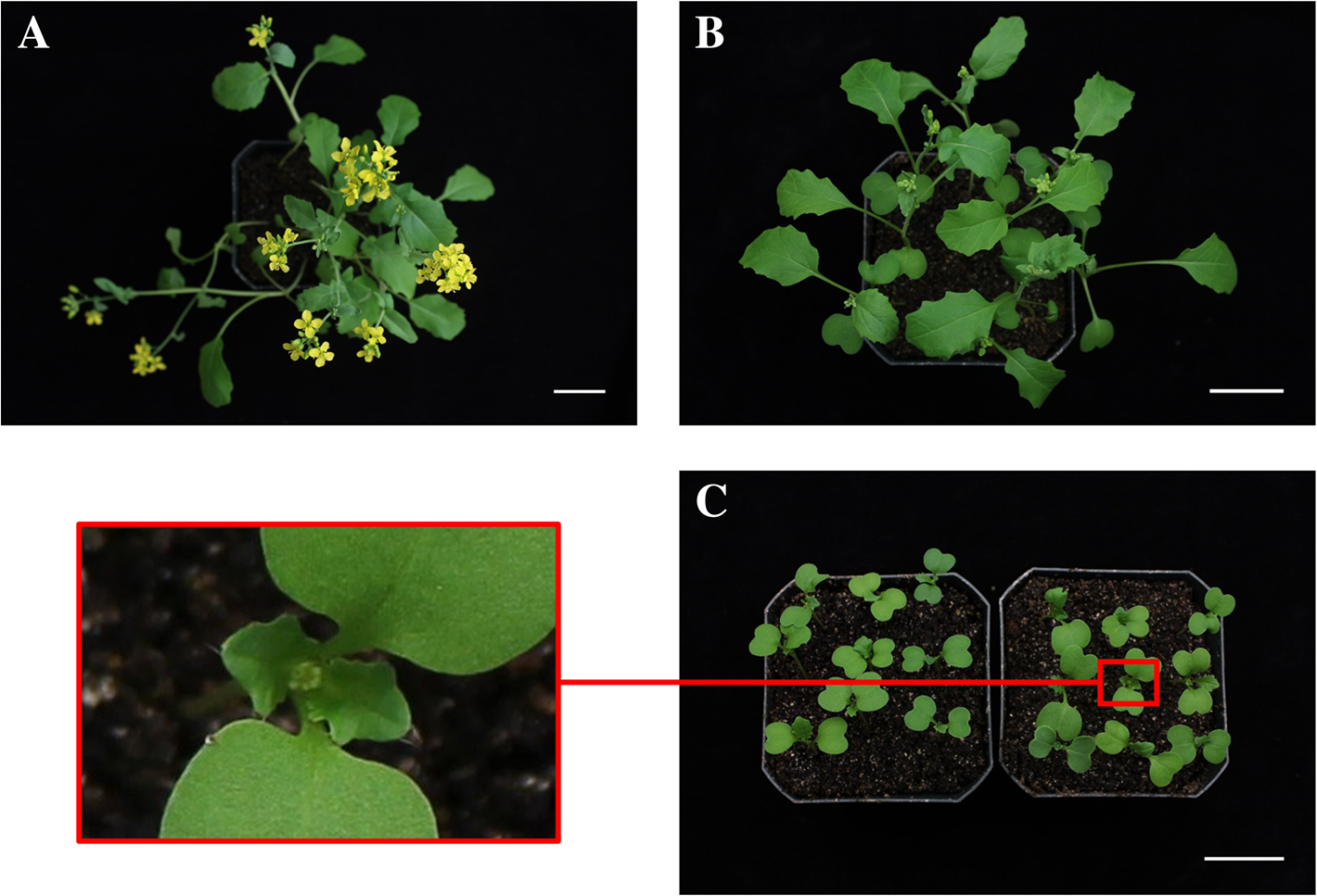
Stages of rapid-cycling *Brassica rapa* chosen to transform. **a** 22-day-old seedlings. **b** 14-day-old seedlings. **c** 8-day-old seedlings. Scale bars, 3 cm

Transformation of RCBr was attempted using both vacuum infiltration and floral dip approaches. *Agrobacterium tumefaciens* strain GV3101(pMP90) carrying binary plasmid p201G was utilized to deliver a 2X35S-GFP DNA construct. To test whether immature floral buds could be transformed and identify the best plant stage for *Agrobacterium* inoculation, different aged plants were tested. In the growth conditions used, 22-day-old plants were at a stage with recently opened flowers and immature buds of various ages (Fig. 3a). 14-day-old plants had larger unopened floral buds and buds < 1 mm in diameter (Fig. 3b), while 8-day-old seedlings were at a stage only containing the putative ideal buds < 1 mm in diameter (Fig. 3c). On the plants from these growth conditions that were more carefully examined, the ~ 1 mm buds on 14-day-old plants were the 4th to 9th bud formed. Hence all plants carried buds of diameter less than 1 mm at the time of inoculation. However, floral dip was not attempted on 22-day-old plants because under our conditions the average number of pods formed per RCBr plant was around 4, but the buds predicted to be transformable on 22-day-old seedlings were 10th or 11th bud formed (Fig. 2). Due to their small size, vacuum infiltration was not used on 8-day-old seedlings. Seeds were harvested for all other treatments, generating a total of over 32,000 candidate T_1_ seeds across all treatments and treatment dates (Table 1).

**Table 1:** Transformation efficiencies for rapid-cycling *Brassica rapa*

Method	Age of Seedlings (d)	Number of Seeds	Number of GFP-tagged T1 Seedlings	Transformation rate
Vacuum infiltration	22	1831	8	0.44%
		763	0	0.00%
		540	0	0.00%
		759	0	0.00%
		507	0	0.00%
		768	1	0.13%
Vacuum infiltration	14	2065	2	0.10%
		2669	4	0.15%
		2928	6	0.20%
Floral dip	14	1437	0	0.00%
		1295	2	0.15%
		775	0	0.00%
		745	1	0.13%
		1653	0	0.00%
		1307	1	0.08%
		1019	1	0.10%
		1585	0	0.00%
		1348	2	0.15%
		1640	0	0.00%
Floral dip	8	1621	1	0.06%
		1722	1	0.06%
		1712	3	0.18%
		529	0	0.00%
		934	0	0.00%
		710	0	0.00%
Overall		32,862	33	0.10%

**Fig. 4:**
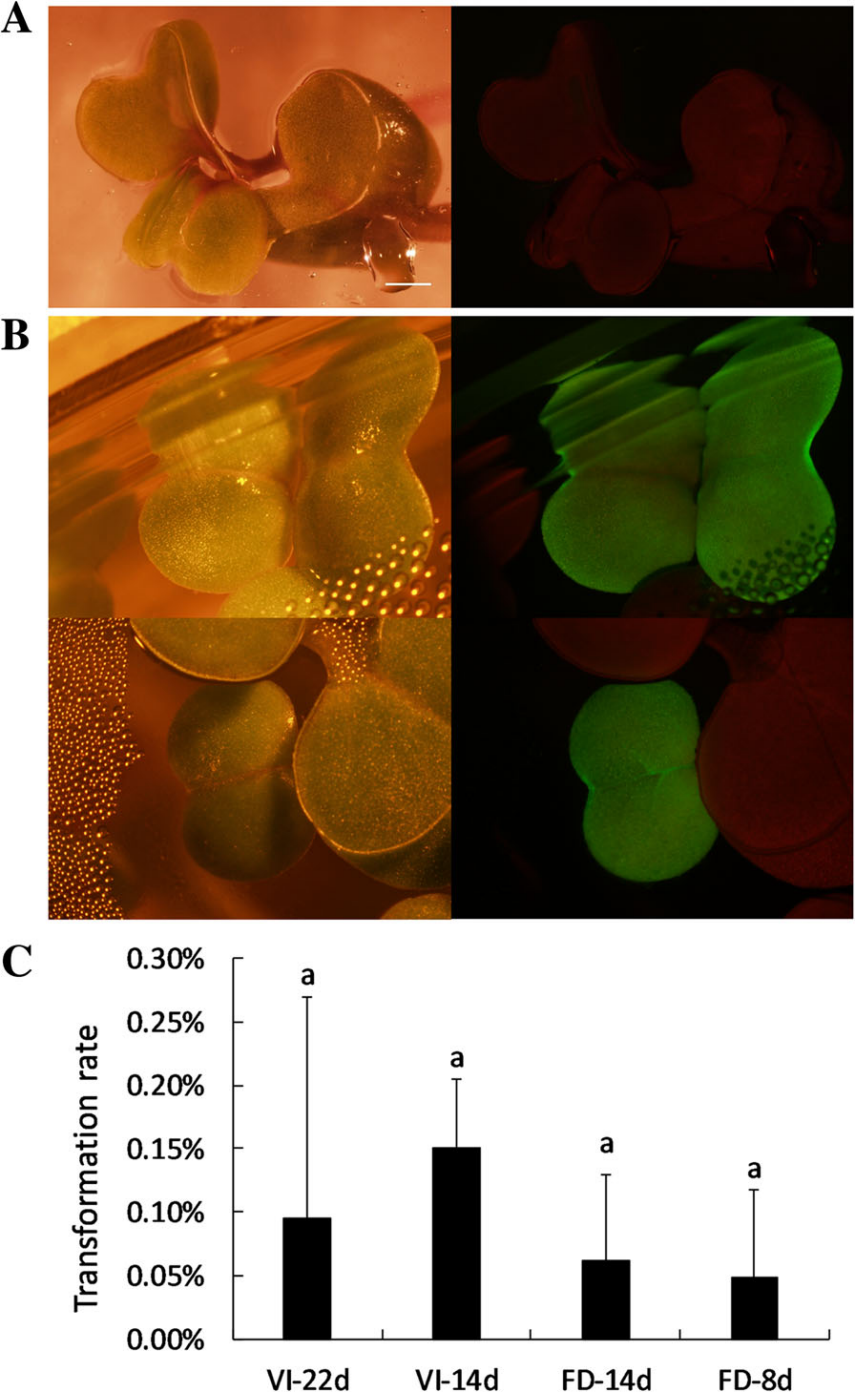
Transformation of rapid-cycling *Brassica rapa*. **a** An example of GFP-negative seedlings. Scale bar, 1 mm. **b** Two examples of GFP-positive T_1_ seedlings. **c** Transformation efficiencies for the different treatments. Mean +/− SE for the data in Table 1. The means are not significantly different (ANOVA, *p* < 0.05). VI, vacuum infiltration; FD, floral dip

T_1_ seedlings were subsequently screened using a fluorescence stereomicroscope 4–5 days after germination on half-strength MS media. GFP-positive plants were found in all four groups (Table 1; Fig. 4b). The results indicate that both the vacuum infiltration and floral dip methods can be used to transform RCBr via inoculation at multiple growth stages. No significant differences in transformation rate were observed between treatments in the present study (Fig. 4c).

### Confirmation of transformation

**Fig. 5:**
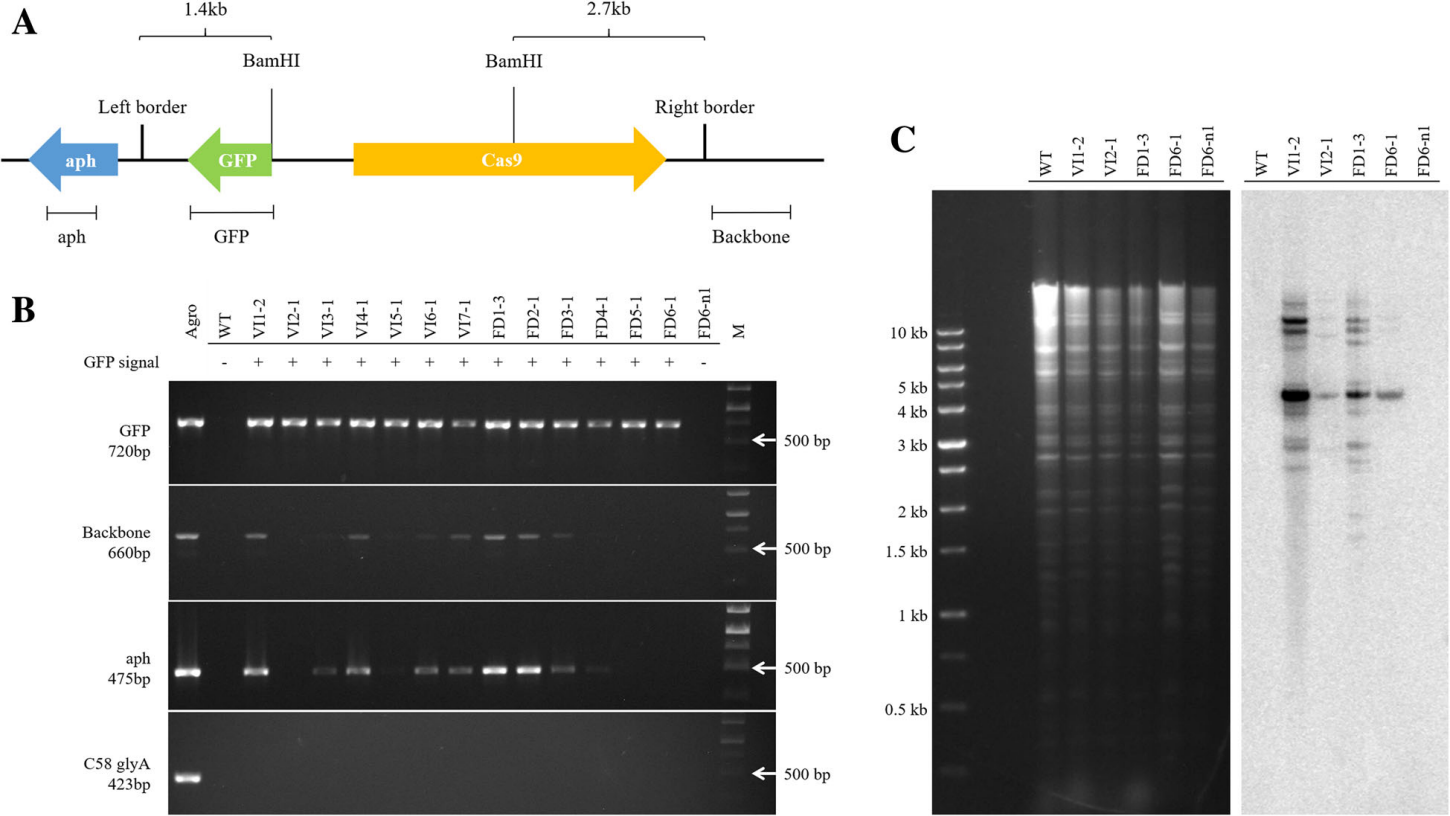
Verification of rapid-cycling *Brassica rapa* transformation. **a** Schematic of T-DNA and flanking region of the circular binary vector p201G, with corresponding aph, GFP and backbone PCR products depicted at bottom. **b** representative results of PCR assays; +, GFP-positive; −, GFP-negative; Agro, *Agrobacterium* strain GV3101(pMP90)(p201G); WT, wild-type RCBr plant; VI, T_2_ from vacuum infiltration; FD, T_2_ from floral dip; M, marker. C58 glyA is an *Agrobacterium* chromosomal gene. **c** Southern blot analysis. Left, electrophoresis gel of *Bam*HI-digested plant genomic DNA; right, Southern blot probed with GFP probe indicated in (**a**)

T_2_ seedlings, from seeds that were obtained after self-fertilization of GFP-positive T_1_ plants, were also screened for GFP signal using a fluorescence stereomicroscope. As expected for seed from hemizygous T_1_ plants, both GFP-positive and -negative plants were found among the T_2_ progeny of GFP-positive T_1_ plants. To further confirm genetic transformation, DNA was extracted from T_2_ plants and PCR and Southern blot assays were performed. Due to concerns that GFP fluorescence could arise from persistent *Agrobacterium*, four primer pairs were used for the PCR assays. Three of primer pairs target *Agrobacterium* DNAs not situated between the T-DNA left border and right border (Fig. 5a). The “aph” and “backbone” primer pairs amplify binary plasmid sequences outside of the left and right borders respectively (Fig. 5a), while the “C58 glyA” primer pair amplifies a portion of the *glyA* gene on the *A. tumefaciens* chromosome [24]. PCR results showed that all GFP-positive T_2_ plants tested produced a GFP amplicon, while WT (non-inoculated) RCBr plants and T_2_ plants lacking green fluorescence were negative for the GFP amplicon (see for example Fig. 5b). Importantly, none of the GFP-positive T_2_ plants tested were positive for *A. tumefaciens glyA* signal. But surprisingly, over half of the plants tested gave a positive signal for the aph or backbone primer pairs, indicating persistent presence of DNA from outside of the T-DNA borders.

Samples from four T_2_ plants that were GFP-positive by PCR assay, one GFP-negative T_2_ plant from a GFP-positive T_1_ plant, and one WT RCBr plant (non-inoculated) were also tested by Southern blot. Genomic DNA was digested with restriction enzyme *Bam*HI, separated by agarose gel electrophoresis, blotted and probed with a 720 bp fragment of *GFP* (see Fig. 5a). Chromosomally integrated T-DNA appearing on the gel blot should be at least 1.4 kb, and should vary in size depending on the variable distance to the next *Bam*HI site, which would in many cases be provided by the flanking RCBr genome at the site of T-DNA integration (Fig. 5a). The observed minimal size of bands on the blot was about 1.5 kb (Fig. 5c). Multiple insertions were evident in the GFP-positive T_2_ plants (Fig. 5c). As is often observed after *Agrobacterium*-mediated plant transformation, some bands were observed that have shared size between independent transformants. These apparently result from intact T-DNA transfer beyond the left border to the next *Bam*HI site in the vector (an expected 10.2 kb band for p201G), and from head-to-tail, tail-to-tail and other repetitive configurations of the integrated T-DNA. Importantly, in three of the four putative transformants, *Bam*HI bands were also detected that were of unique size only to that transformant. The results provide strong evidence that the tested GFP-positive T_2_ plants were genuine stable transformants.

In total, we obtained 33 GFP-positive T_1_ plants in the present study (Table 1). Ten of them were sterile or produced low quality seeds that did not germinate, while T_2_ families were built from the other 23 T_1_ events. GFP screening results showed that all of these T_2_ families contained GFP-positive plants. The T_2_ samples used in the PCR assay of Fig. 5b were from 13 different T_1_ plants (FD6–1 and FD6-n1 were from the same T_1_ plant). Hence evidence for the heritability of the transformation events, from T_1_ transformant to T_2_ progeny, was obtained for all of the GFP-positive T_1_ plants that produced viable T_2_ seed.

## Discussion

Different genetic strains or subspecies of *Brassica rapa* exhibit strikingly different growth forms and uses (e.g., pakchoi, turnip, oilseed field mustard). Rapid-cycling *Brassica rapa* are very widely used in science education, and also have utility for scientific research. Genetic transformation of RCBr had not been reported. Although transformation methods that use tissue culture (transgenic callus production and plant regeneration) have been reported for *Brassica rapa*, they are difficult. Given past successes with *in planta* germline transformation not only with *Arabidopsis* but also with other Brassicaceae including *Brassica rapa* ssp. *chinensis* (pakchoi or bok choy), it was logical to attempt transformation of RCBr using vacuum infiltration or floral dip methods. Across all the treatment methods in this study, a transformation success rate of 0.1% was achieved (33 out of 32,862 candidate T_1_ seeds tested). This is approximately ten-fold lower than the success rates commonly obtained with *Arabidopsis* but given the low amount of overall labor involved, it is well within range for a practically useful transformation method.

Kanamycin is widely used in plant transformation systems to select transformants, but high densities of seeds during selection have been shown to decrease the selective efficiency [25]. In our study, two different densities of wild-type RCBr were tested for kanamycin susceptibility and green seedlings were still found on plates even at very low densities using kanamycin at up to 200 mg/L (Fig. 1). This indicated that RCBr are less sensitive to kanamycin. Takasaki et al. [26] used *Agrobacterium*-mediated tissue culture to transform *Brassica rapa* ssp. *chinensis* and could not avoid escaped non-transgenic shoots using 50 mg/L kanamycin selection. Kuvshinov et al. [27] had a similar issue while transforming *Brassica rapa* ssp. *oleifera*, that the portion of escaped shoots was 90% under kanamycin selection. However, explant tissue of *Brassica rapa* showed acceptable sensitivity to kanamycin [28]. These studies suggest that the kanamycin sensitivities among *Brassica rapa* plants are variable. Although GFP worked well in our RCBr transformation system, it is a screenable marker that requires researchers to be the “selective” agent. Considering this aspect, other selectable markers could be tested on RCBr in future studies.

Ovules are known to be the primary target of *Agrobacterium*-mediated vacuum infiltration and floral dip transformation in *Arabidopsis* [21–23]. Providing *Agrobacterium* with ready access to developing ovules in the locule interior is likely to be a key aspect for success with *in planta* transformation of other plants as well. Desfeux et al. [23] found that transformation failed with floral buds inoculated past the stage at which the stigmatic cap closes the locule, and found that the rate of transformation of an *Arabidopsis* CRABS-CLAW mutant that has unsealed carpels was 6-fold higher than the transformation rate of wild-type plants. Dissection work was executed in our study to identify buds with unclosed carpels. We found that the diameter of such buds was less than 1 mm (Fig. 2), which was similar to the result that the pakchoi flowers with GUS-stained ovules after *Agrobacterium* inoculation were about 0.5–1.0 mm in diameter and had an open ovary when vacuum infiltration was carried out [15]. Although Xu et al. [15] found GUS stained pollen in the same sample with stained ovules, they did not determine if either or both were the source of successfully transgenic plants. In *Arabidopsis*, Ye et al. [21] found mature pollen expressing GUS after *Agrobacterium* infiltration, while Desfeux et al. [23] did not find such pollen after floral dipping. However, neither of them could get transformants from crossing when inoculated plants served as pollen donors and non-inoculated plants served as pollen recipients [21, 23]. Recently, it was reported that prairie gentian (*Eustoma grandifloru*; also called lisianthus) was successfully transformed via the floral dip method at a post-anthesis stage when ovaries were partially sealed, while in these flowers a stylar channel remained open [18]. Another study recently reported tomato transformation by floral bud injection [17], another approach to providing *Agrobacterium* with access to ovules. We took care to only inoculate plants carrying immature flower buds (< 1 mm), but were surprised that we did not observe significant differences in transformation frequency between plants with only immature buds or plants already carrying open flowers as well as immature buds. Additional studies in multiple laboratories will be required to determine if transformation rates are more favorable for certain plant growth stages or other treatment regimes. In practical terms, the fecundity of plants is another factor influencing the transformation success, especially for self-infertile accessions.

With *Agrobacterium* mediated transformation using binary vectors it is common that backbone sequences (DNA outside of the left and right borders) can be integrated into the plant genome [29]. The frequencies of this phenomenon were over 60% in transgenic tobacco [30], strawberry [31] and wheat [32]. PCR analysis from our research presented a similar result that many of the examined plants had backbone sequence integration events (Fig. 5b). Strikingly, the entire vector sequence can be incorporated into the plant DNA [33, 34]. This apparently happened in our study as well, as we observed multiple T_2_ plants that were PCR-positive for all three of the vector sequence primer pairs (GFP, aph and backbone). Southern blot evidence similarly showed some T-DNAs that extended past the left border to include the full 10.2 kb *Bam*HI fragment of the p201G vector (Fig. 5c). The Southern blot lanes shared other bands among transformants, especially between entirely independent VI1–2 and FD1–3, suggesting that direct or inverted T-DNA repeats had been integrated into one locus, while the bands of unique size revealed independent insertions (Fig. 5c). Multicopy insertions, which have the potential to cause transgene silencing [35], are often less desirable. Most of the tested GFP-positive plants in our study had this issue. To address the problem, Oltmanns et al. [36] have suggested launching T-DNA from the *Agrobacterium* chromosome, which could also reduce the backbone sequence integration. De Paepe et al. [37] reported that using a T-DNA vector with one *loxP* site to transform *CRE*-expressing *Arabidopsis* would increase the frequency of single-copy events. *Arabidopsis* researchers have reduced the frequency of multicopy inserts by switching to less efficient *Agrobacterium* strains. These and other refinements remain to be explored in RCBr, for which successful vacuum infiltration and floral dip transformation have now been demonstrated.

## Conclusion

A transformation system for RCBr was established in this study. This will promote development of new biology teaching tools as well as basic biology research on *Brassica rapa*.

## Methods

### Plant growth and pollination

Rapid-cycling *Brassica rapa* seeds (standard line, RcBC 1-33, available from the Rapid-Cycling Brassica Collection, University of Wisconsin - Madison, www.rcbc.wisc.edu) were kindly provided by Dr. Paul Williams (University of Wisconsin – Madison, USA). Seeds were sown 9 per pot in a soil mixture of Sun Gro® (Sun Gro Horticultural, Agawam, MA, USA) propagation mix and vermiculite (1:1). For vacuum infiltration experiments, pots were covered with tulle fabric (plastic mesh with pore diameter approximately 1 mm) at planting, to prevent soil from falling into *Agrobacterium* inoculum during infiltration. Plants were grown at 24 °C under a 16-h light/8-h dark photoperiod. After inoculation with *Agrobacterium*, flowers of T_0_ plants were manually cross-fertilized every day (for at least 1 week) on the most recently opened flowers, using a bee-stick (the body of a dead bee glued to a toothpick) carrying mixed pollen from multiple inoculated RCBr plants. Self-fertilization was performed on buds of T_1_ plants to generate T_2_ seeds. For self-fertilization, forceps were used to remove sepals and petals and expose the young stigma on newly developing flowers. Stamens were collected from opened flowers of the same plant and used for pollination. Forceps were soaked in 70% ethanol before each self-fertilization to avoid pollen contamination.

### *Agrobacterium tumefaciens* strain, culture and inoculation

*Agrobacterium tumefaciens* strain GV3101(pMP90) carrying binary vector p201G was used in all experiments [38]. p201G carries a 2X35S promoter-mGFP5-*nos* terminator gene construct [39]. *Agrobacterium* was diluted in LB medium (1:100) with 25 mg/L kanamycin and 50 mg/L rifampicin, and cultured overnight at 28 °C, 200 rpm. Bacteria were collected by centrifugation at 5000 rpm for 10 min at room temperature and resuspended with 5% sucrose and 200 μL/L Silwet L-77 to a final OD_600_ of 0.8–1.0. Vacuum infiltration or floral dip were performed following the procedure described by Clough and Bent [20]. For vacuum infiltration, at least 800 mL of inoculum was used. Above-soil portions of plants were inverted into the *Agrobacterium* solution in a desiccator jar which was then attached to a vacuum pump via rubber tube. A vacuum was established in the desiccator jar until the infiltration solution had formed bubbles for approximately 10 s, at which time the desiccator jar was opened to atmospheric pressure by removal of the rubber tube, causing infiltration of the *Agrobacterium* solution into the immersed plant tissues. For floral dip, about 3 mL of *Agrobacterium* solution was used by pipetting onto multiple flower buds. After either form of inoculation, plants were placed in a plastic tray and covered by a plastic dome to maintain elevated humidity. Plants were then maintained in a low-light environment for 16–24 h and then uncovered and returned to the growth chamber in which they had previously been cultivated. For each treatment, more than 20 plants were inoculated.

### Bud dissection

Flowers and buds were taken from the same inflorescence. Fine forceps were used to separate sepals and petals. A stereomicroscope was employed to look for unsealed carpels where the stigma had not fully formed. Photos were taken with an attached Olympus DP73 camera. Pre-dissection diameters were measured by Fiji software.

### Kanamycin sensitivity testing or GFP screening

RCBr seeds were surface-sterilized for around 20 h under chlorine gas using the vapor-phase method [20]. For kanamycin sensitivity testing, surface-sterilized wild-type seeds were sown onto half strength Murashige and Skoog agar media containing 0, 50, 100, 150 or 200 mg/L kanamycin. The densities were 49 or 9 seeds per plate. For GFP screening, sterilized seeds were plated onto half strength MS media without antibiotic agents. Seedlings were grown in the same environment described above. To detect GFP signal, 4- to 7-day-old seedlings were screened using a Leica MZ FLIII fluorescence stereomicroscope with GFP2 (GFP plus) filter. Photos were taken with an attached Olympus DP73 camera.

### PCR and Southern blots

Total genomic DNA of T_2_ plants was isolated via a CTAB method. GoTaq® Green Master Mix (Promega) was used in PCR assays. Primers are listed in Additional file 1. The PCR program included pre-denaturation at 95 °C for 3 min, followed by 30 cycles of denaturing at 95 °C for 30 s, annealing at 57 °C for 30 s and extension at 72 °C for 45 s, and a final extension at 72 °C for 5 min. The products were analyzed by electrophoresis in 1.0% agarose-ethidium bromide gels.

Southern blot experiments were conducted as in Current Protocols in Molecular Biology [40]. Approximately 10 μg of genomic DNA of each sample was digested with the restriction enzyme *Bam*HI, resolved by electrophoresis in a 0.7% agarose gel, transferred to a Hybond™-N membrane, crosslinked by baking at 80 °C for 2 h. Blots were then probed with a ^32^P-labeled *GFP* gene fragment (Additional file 1) and imaged using a Typhoon™ FLA 9000 gel imaging scanner.

## Additional files

Additional file 1: Primers for PCR assay. (XLSX 9 kb)

Additional file 2: Wild-type rapid-cycling *Brassica rapa* with no GFP expression. (TIF 525 kb)

## Data Availability

All data generated or analysed during this study are included in this published article.

## Abbreviations

FD: Floral dip
GFP: Green fluorescent protein
RCBr: Rapid-cycling of *Brassica rapa*
VI: Vacuum infiltration
