# Supplementary figures and images for: Agrobacterium-mediated vacuum infiltration and floral dip transformation of rapid-cycling Brassica rapa
Source: BMC Plant Biol. 2019 Jun 10;19:246. doi: 10.1186/s12870-019-1843-6 (PMC6558690; doi:10.1186/s12870-019-1843-6)

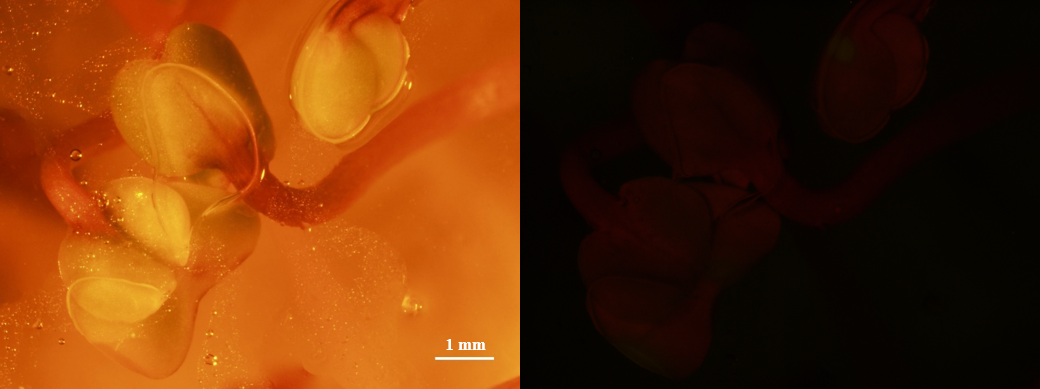

Supplement: Supplementary file 2 — Wild-type rapid-cycling Brassica rapa with no GFP expression. (TIF 525 kb) [file 12870_2019_1843_MOESM2_ESM.tif]
